# 2,2′-Bi[6,6′-dimethyl­dibenzo[*d*,*f*][1,3]dioxepine]

**DOI:** 10.1107/S1600536808009094

**Published:** 2008-04-10

**Authors:** Hai-Quan Zhang, Bao-Li, Guang-Di Yang, Yu-Guang Ma

**Affiliations:** a State Key Laboratory of Metastable Materials Science and Technology, Yanshan University, Qinhuangdao 066004, People’s Republic of China; b State Key Laboratory of Supramolecular Structure and Materials, Jilin University, Changchun 130012, People’s Republic of China

## Abstract

The title compound, C_30_H_26_O_4_, is a dimer of 6,6′-dimethyl­dibenzo[*d*,*f*][1,3]dioxepine linked by formation of a C—C bond in the *para* position with respect to one O atom. The dimer is arranged around an inversion centre. As is usually observed in related compounds, the dibenzo group is twisted, the two benzene rings making a dihedral angle of 41.56 (9)°. The seven-membered ring exhibits a twisted conformation.

## Related literature

For related literature, see: Colon & Kelsey (1986 ▶); McCullough (1998 ▶); Samdal & Bastiansen (1985 ▶); Silcox Yoder & Zuckerman (1967 ▶); Suzuki (1959 ▶); Harada *et al.* (1994 ▶, 1997 ▶); Pajunen *et al.* (1996 ▶).
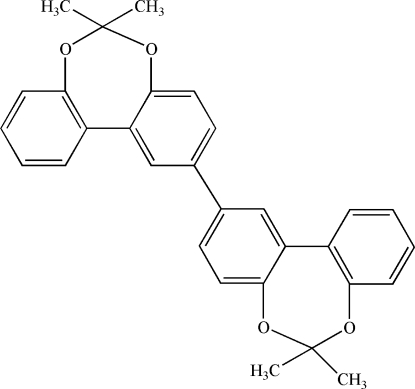


## Experimental

### 

#### Crystal data

C_30_H_26_O_4_
*M* *_r_* = 450.51
Monoclinic, 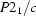

*a* = 13.2938 (16) Å
*b* = 7.3200 (17) Å
*c* = 12.7067 (11) Å
β = 103.61 (3)°
*V* = 1201.8 (4) Å^3^
*Z* = 2
Mo *K*α radiation
μ = 0.08 mm^−1^
*T* = 293 (2) K
0.18 × 0.13 × 0.13 mm

#### Data collection

Rigaku R-AXIS RAPID diffractometer
Absorption correction: multi-scan (*ABSCOR*; Higashi, 1995 ▶) *T*_min_ = 0.985, *T*_max_ = 0.990
5035 measured reflections
2712 independent reflections
1170 reflections with *I* > 2σ(*I*)
*R*_int_ = 0.072

#### Refinement

*R*[*F* ^2^ > 2σ(*F* ^2^)] = 0.037
*wR*(*F* ^2^) = 0.126
*S* = 0.81
2712 reflections
156 parameters
H-atom parameters constrained
Δρ_max_ = 0.13 e Å^−3^
Δρ_min_ = −0.17 e Å^−3^

### 

Data collection: *RAPID-AUTO* (Rigaku, 1998 ▶); cell refinement: *RAPID-AUTO*; data reduction: *CrystalStructure* (Molecular Structure Corporation & Rigaku , 2002 ▶); program(s) used to solve structure: *SHELXS97* (Sheldrick, 2008 ▶); program(s) used to refine structure: *SHELXL97* (Sheldrick, 2008 ▶); molecular graphics: *ORTEPIII* (Burnett & Johnson, 1996 ▶) and *ORTEP-3 for Windows* (Farrugia, 1997 ▶); software used to prepare material for publication: *SHELXL97*.

## Supplementary Material

Crystal structure: contains datablocks global, I. DOI: 10.1107/S1600536808009094/dn2334sup1.cif

Structure factors: contains datablocks I. DOI: 10.1107/S1600536808009094/dn2334Isup2.hkl

Additional supplementary materials:  crystallographic information; 3D view; checkCIF report

## Figures and Tables

**Fig. 1.**
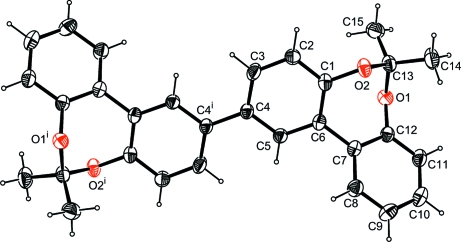
Molecular view of the title compound with the atom-labelling scheme. Displacement ellipsoids are drawn at the 30% probability level. H atoms are represented as small spheres of arbitrary radii. [Symmetry code: (i) 1-x, 1-y, 1-z].

## supplementary crystallographic information

### Comment

The inter-ring twisted angles in biphenyl compounds are widely studied because changing of conformations of the conjugated molecules affect the optical and electronic properties significantly (McCullough, 1998). Most of biphenyl derivatives showed the twisting conformation in their gas phase or solution, while in crytal a few of them have planar or nearly planar conformation at room temperature (Samdal *et al.*, 1985; Suzuki, 1959). Strong face-to-face interactions induced planar conformation of solid biphenyl.

The title compound, C_30_H_26_O_4_, is a dimer of the 6,6'-dimethyl-dibenzo [d,f][1,3]dioxepin linked by formation of a C-C bond in para position with respect to one O atom (Fig. 1). This dimer is arranged around inversion centre. As usually observed in related compounds ( Pajunen *et al.*, 1996; Harada *et al.*, 1994,1997), the dibenzo group is twisted with the two benzene rings making a dihedral angle of 41.56?(9)°.

### Experimental

Synthesis approach of dimeric 6,6'-dimethyl-dibenzo [d,f][1,3]dioxepine was described as follows: The precursor 5-bromo-2,2'-dihydroxybiphenyl was synthesized by directly brominating of dihydroxybiphenyl. The 2-bromo-6,6'-dimethyl- dibenzo [d, f][1, 3] dioxepine was obtained according to the previously reportable reaction condition (Silcox Yoder & Zuckerman, 1967). Finally, the *meta*-linked dimeric 6,6'-dimethyl-dibenzo [d, f][1, 3] dioxepine was prepared according to the typical Yamamoto condition (Colon *et al.*, 1986). Crystals suitable for single-crystal X-ray diffraction were grown by slow evaporation of a ethanol solution.

### Refinement

All H atoms were fixed geometrically and treated as riding with C—H = 0.93 Å (aromatic) and 0.96 Å (methyl) with U_iso_(H) = 1.2U_eq_(aromatic) or U_iso_(H) = 1.5U_eq_(methyl).

### Crystal data

**Table:**

C_30_H_26_O_4_	*F*_000_ = 476
*M**_r_* = 450.51	*D*_x_ = 1.245 Mg m^−^^3^
Monoclinic, *P*2_1_/*c*	Mo *K*α radiation λ = 0.71073 Å
Hall symbol: -P 2ybc	Cell parameters from 2961 reflections
*a* = 13.2938 (16) Å	θ = 5.1–54.6º
*b* = 7.3200 (17) Å	µ = 0.08 mm^−^^1^
*c* = 12.7067 (11) Å	*T* = 293 (2) K
β = 103.61 (3)º	Block, colorless
*V* = 1201.8 (4) Å^3^	0.18 × 0.13 × 0.13 mm
*Z* = 2	

### Data collection

**Table:**

Rigaku R-AXIS RAPID diffractometer	2712 independent reflections
Radiation source: fine-focus sealed tube	1170 reflections with *I* > 2σ(*I*)
Monochromator: graphite	*R*_int_ = 0.072
*T* = 293(2) K	θ_max_ = 27.5º
ω scans	θ_min_ = 1.6º
Absorption correction: multi-scan(ABSCOR; Higashi, 1995)	*h* = −17→16
*T*_min_ = 0.985, *T*_max_ = 0.990	*k* = −9→9
5035 measured reflections	*l* = 0→16

### Refinement

**Table:**

Refinement on *F*^2^	Secondary atom site location: difference Fourier map
Least-squares matrix: full	Hydrogen site location: inferred from neighbouring sites
*R*[*F*^2^ > 2σ(*F*^2^)] = 0.037	H-atom parameters constrained
*wR*(*F*^2^) = 0.126	*w* = 1/[σ^2^(*F*_o_^2^) + (0.0601*P*)^2^] where *P* = (*F*_o_^2^ + 2*F*_c_^2^)/3
*S* = 0.81	(Δ/σ)_max_ < 0.001
2712 reflections	Δρ_max_ = 0.13 e Å^−^^3^
156 parameters	Δρ_min_ = −0.16 e Å^−^^3^
Primary atom site location: structure-invariant direct methods	Extinction correction: none

### Special details

**Table:**

Geometry. All esds (except the esd in the dihedral angle between two l.s. planes) are estimated using the full covariance matrix. The cell esds are taken into account individually in the estimation of esds in distances, angles and torsion angles; correlations between esds in cell parameters are only used when they are defined by crystal symmetry. An approximate (isotropic) treatment of cell esds is used for estimating esds involving l.s. planes.
Refinement. Refinement of *F*^2^ against ALL reflections. The weighted *R*-factor *wR* and goodness of fit *S* are based on *F*^2^, conventional *R*-factors *R* are based on *F*, with *F* set to zero for negative *F*^2^. The threshold expression of *F*^2^ > σ(*F*^2^) is used only for calculating *R*-factors(gt) *etc*. and is not relevant to the choice of reflections for refinement. *R*-factors based on *F*^2^ are statistically about twice as large as those based on *F*, and *R*- factors based on ALL data will be even larger.

### Fractional atomic coordinates and isotropic or equivalent isotropic displacement parameters (Å^2^)

**Table:**

	*x*	*y*	*z*	*U*_iso_*/*U*_eq_	
C1	0.65924 (15)	0.3273 (3)	0.74573 (16)	0.0426 (5)	
C2	0.59655 (17)	0.4785 (3)	0.74799 (17)	0.0541 (6)	
H2	0.5968	0.5361	0.8133	0.065*	
C3	0.53331 (16)	0.5431 (3)	0.65184 (17)	0.0554 (6)	
H3	0.4909	0.6434	0.6542	0.066*	
C4	0.53170 (14)	0.4619 (3)	0.55227 (16)	0.0400 (5)	
C5	0.59453 (15)	0.3071 (3)	0.55358 (16)	0.0411 (5)	
H5	0.5941	0.2488	0.4884	0.049*	
C6	0.65776 (15)	0.2372 (3)	0.64907 (16)	0.0394 (5)	
C7	0.72262 (15)	0.0713 (3)	0.65022 (17)	0.0418 (5)	
C8	0.68640 (17)	−0.0820 (3)	0.58720 (19)	0.0523 (6)	
H8	0.6193	−0.0829	0.5441	0.063*	
C9	0.74995 (19)	−0.2334 (3)	0.5884 (2)	0.0610 (7)	
H9	0.7250	−0.3349	0.5463	0.073*	
C10	0.85074 (19)	−0.2339 (3)	0.6522 (2)	0.0605 (7)	
H10	0.8932	−0.3348	0.6517	0.073*	
C11	0.88765 (17)	−0.0849 (3)	0.71621 (18)	0.0524 (6)	
H11	0.9546	−0.0854	0.7595	0.063*	
C12	0.82413 (15)	0.0658 (3)	0.71547 (16)	0.0409 (5)	
C13	0.82810 (16)	0.2702 (3)	0.86599 (17)	0.0477 (6)	
C14	0.86854 (19)	0.1391 (4)	0.95822 (19)	0.0705 (8)	
H14A	0.8475	0.0170	0.9356	0.106*	
H14B	0.8411	0.1718	1.0191	0.106*	
H14C	0.9427	0.1453	0.9785	0.106*	
C15	0.86209 (18)	0.4649 (4)	0.89127 (19)	0.0651 (7)	
H15A	0.9360	0.4726	0.9031	0.098*	
H15B	0.8416	0.5042	0.9552	0.098*	
H15C	0.8303	0.5420	0.8315	0.098*	
O1	0.86503 (10)	0.2224 (2)	0.77162 (11)	0.0462 (4)	
O2	0.71592 (11)	0.2534 (2)	0.84347 (11)	0.0493 (4)	

### Atomic displacement parameters (Å^2^)

**Table:**

	*U*^11^	*U*^22^	*U*^33^	*U*^12^	*U*^13^	*U*^23^
C1	0.0361 (12)	0.0505 (14)	0.0436 (13)	0.0058 (10)	0.0139 (10)	0.0117 (11)
C2	0.0607 (14)	0.0664 (17)	0.0377 (12)	0.0200 (13)	0.0166 (11)	0.0001 (11)
C3	0.0576 (15)	0.0582 (16)	0.0529 (14)	0.0273 (12)	0.0183 (12)	0.0067 (12)
C4	0.0332 (11)	0.0441 (13)	0.0430 (12)	0.0054 (9)	0.0098 (9)	0.0036 (10)
C5	0.0393 (12)	0.0404 (13)	0.0430 (12)	0.0015 (10)	0.0089 (10)	−0.0004 (10)
C6	0.0349 (11)	0.0384 (13)	0.0457 (12)	0.0019 (9)	0.0110 (10)	0.0073 (11)
C7	0.0384 (12)	0.0401 (13)	0.0483 (12)	0.0036 (10)	0.0129 (10)	0.0064 (10)
C8	0.0451 (13)	0.0463 (15)	0.0636 (15)	0.0007 (12)	0.0090 (11)	0.0006 (12)
C9	0.0665 (18)	0.0381 (15)	0.0815 (19)	0.0002 (12)	0.0234 (15)	−0.0035 (13)
C10	0.0590 (16)	0.0491 (17)	0.0763 (17)	0.0181 (12)	0.0218 (14)	0.0135 (14)
C11	0.0453 (13)	0.0566 (16)	0.0551 (14)	0.0131 (12)	0.0114 (11)	0.0078 (12)
C12	0.0403 (12)	0.0411 (13)	0.0428 (12)	0.0027 (10)	0.0127 (10)	0.0069 (10)
C13	0.0401 (13)	0.0612 (17)	0.0413 (12)	0.0084 (11)	0.0085 (10)	0.0063 (12)
C14	0.0671 (16)	0.090 (2)	0.0531 (15)	0.0251 (15)	0.0109 (13)	0.0220 (14)
C15	0.0653 (16)	0.074 (2)	0.0551 (15)	0.0012 (14)	0.0116 (13)	−0.0098 (13)
O1	0.0390 (8)	0.0568 (10)	0.0443 (8)	0.0003 (7)	0.0127 (7)	0.0004 (8)
O2	0.0426 (9)	0.0647 (11)	0.0422 (8)	0.0092 (7)	0.0131 (7)	0.0162 (8)

### Geometric parameters (Å, °)

**Table:**

C1—C2	1.389 (3)	C9—H9	0.9300
C1—C6	1.390 (3)	C10—C11	1.380 (3)
C1—O2	1.401 (2)	C10—H10	0.9300
C2—C3	1.393 (3)	C11—C12	1.388 (3)
C2—H2	0.9300	C11—H11	0.9300
C3—C4	1.394 (3)	C12—O1	1.391 (2)
C3—H3	0.9300	C13—O1	1.442 (3)
C4—C5	1.405 (3)	C13—O2	1.456 (3)
C4—C4^i^	1.503 (4)	C13—C15	1.507 (3)
C5—C6	1.400 (3)	C13—C14	1.512 (3)
C5—H5	0.9300	C14—H14A	0.9600
C6—C7	1.488 (3)	C14—H14B	0.9600
C7—C8	1.397 (3)	C14—H14C	0.9600
C7—C12	1.408 (3)	C15—H15A	0.9600
C8—C9	1.391 (3)	C15—H15B	0.9600
C8—H8	0.9300	C15—H15C	0.9600
C9—C10	1.393 (3)		
			
C2—C1—C6	120.82 (19)	C11—C10—H10	120.0
C2—C1—O2	119.3 (2)	C9—C10—H10	120.0
C6—C1—O2	119.53 (19)	C10—C11—C12	119.5 (2)
C1—C2—C3	119.6 (2)	C10—C11—H11	120.2
C1—C2—H2	120.2	C12—C11—H11	120.2
C3—C2—H2	120.2	C11—C12—O1	119.18 (18)
C2—C3—C4	121.9 (2)	C11—C12—C7	121.6 (2)
C2—C3—H3	119.0	O1—C12—C7	118.90 (19)
C4—C3—H3	119.0	O1—C13—O2	110.46 (15)
C3—C4—C5	116.81 (19)	O1—C13—C15	105.26 (18)
C3—C4—C4^i^	122.0 (2)	O2—C13—C15	111.31 (18)
C5—C4—C4^i^	121.2 (2)	O1—C13—C14	111.23 (19)
C6—C5—C4	122.6 (2)	O2—C13—C14	105.06 (19)
C6—C5—H5	118.7	C15—C13—C14	113.6 (2)
C4—C5—H5	118.7	C13—C14—H14A	109.5
C1—C6—C5	118.23 (19)	C13—C14—H14B	109.5
C1—C6—C7	119.41 (18)	H14A—C14—H14B	109.5
C5—C6—C7	122.36 (19)	C13—C14—H14C	109.5
C8—C7—C12	117.9 (2)	H14A—C14—H14C	109.5
C8—C7—C6	121.97 (18)	H14B—C14—H14C	109.5
C12—C7—C6	120.12 (19)	C13—C15—H15A	109.5
C9—C8—C7	120.5 (2)	C13—C15—H15B	109.5
C9—C8—H8	119.8	H15A—C15—H15B	109.5
C7—C8—H8	119.8	C13—C15—H15C	109.5
C8—C9—C10	120.5 (2)	H15A—C15—H15C	109.5
C8—C9—H9	119.8	H15B—C15—H15C	109.5
C10—C9—H9	119.8	C12—O1—C13	117.27 (16)
C11—C10—C9	120.0 (2)	C1—O2—C13	117.00 (16)

Symmetry codes: (i) −*x*+1, −*y*+1, −*z*+1.

## References

[bb1] Burnett, M. N. & Johnson, C. K. (1996). *ORTEPIII* Report ORNL-6895. Oak Ridge National Laboratory, Oak Ridge, Tennessee, USA.

[bb2] Colon, I. & Kelsey, D. R. (1986). *J. Org. Chem* **51**, 2627–2637.

[bb3] Farrugia, L. J. (1997). *J. Appl. Cryst.* **30**, 565.

[bb4] Harada, T., Ueda, S., Tuyet, T. M. T., Inoue, A., Fujita, K., Takeuchi, M., Ogawa, N., Oku, A. & Shiro, M. (1997). *Tetrahedron*, **53**, 16663–16678.

[bb5] Harada, T., Ueda, S., Yoshida, T., Inoue, A., Takeuchi, M., Ogawa, N., Oku, A. & Shiro, M. (1994). *J. Org. Chem* **59**, 7575–7576.

[bb6] Higashi, T. (1995). *ABSCOR* Rigaku Corporation, Tokyo, Japan.

[bb7] McCullough, R. D. (1998). *Adv. Mater* **10**, 93–116.

[bb8] Molecular Structure Corporation & Rigaku (2002). *CrystalStructure* MSC, The Woodlands, Texas, USA, and Rigaku Corporation, Tokyo, Japan.

[bb9] Pajunen, A., Karhunen, P. & Brunow, G. (1996). *Acta Cryst.* C**52**, 1815–1817.

[bb10] Rigaku (1998). *RAPID-AUTO* Rigaku Corporation, Tokyo, Japan.

[bb11] Samdal, S. & Bastiansen, O. (1985). *J. Mol. Struct* **128**, 115–125.

[bb12] Sheldrick, G. M. (2008). *Acta Cryst.* A**64**, 112–122. 10.1107/S0108767307043930 18156677

[bb13] Silcox Yoder, C. M. & Zuckerman, J.J. (1967). *J. Heterocycl. Chem* **4**, 166–167.

[bb14] Suzuki, H. (1959). *Bull. Chem. Soc. Jpn*, **32**, 1340–1350.

